# Examining the association between serum IgG of oral bacteria and metabolic syndrome

**DOI:** 10.3389/fmed.2022.899063

**Published:** 2022-07-22

**Authors:** Zhe-Yu Yang, Wen-Hui Fang, Chia-Chun Kao, Wei-Liang Chen

**Affiliations:** ^1^Division of Family Medicine, Department of Family and Community Medicine, Tri-Service General Hospital, School of Medicine, Taipei, Taiwan; ^2^National Defense Medical Center, School of Medicine, Taipei, Taiwan; ^3^Division of Geriatric Medicine, Department of Family and Community Medicine, Tri-Service General Hospital Taipei, Taipei, Taiwan; ^4^Department of Medical Education, Taipei Veterans General Hospital, Taipei, Taiwan; ^5^Department of Biochemistry, National Defense Medical Center, Taipei, Taiwan

**Keywords:** periodontitis, periodontal bacteria, metabolic syndrome, EPI—epidemiology, multivariate analysis

## Abstract

**Aim:**

This investigation explored the relationship between oral bacteria and metabolic syndrome (METS).

**Materials and Methods:**

There were 4,882 subjects enrolled in this cross-sectional study from the NHANES III database. The severity of periodontitis was classified into mild, moderate and severe. We measured oral bacterial antibodies. We examined the relationship between serum immunoglobulin G (IgG) antibodies of oral bacteria and METS *via* performing multivariate regression analysis. Mediation analysis of oral bacteria on the correlation between periodontitis and METS was also executed.

**Results:**

After adjusting for covariates, the serum IgG antibodies of *P. nigrescens, E. corrodens*, and *E. nodatum* were associated with the presence of METS (*p* = 0.006, *p* = 0.014 and *p* = 0.018, respectively). Furthermore, serum IgG antibodies of *P. intermedia, T. forsythia* and *V. parvula* were positively associated with the presence of METS (*p* = 0.001, *p* = 0.011, and *p* = 0.002, respectively) and ≥4 features of METS (*p* = 0.019, *p* = 0.025, and *p* = 0.02, respectively). *P. intermedia* IgG mediated 11.2% of the relationship between periodontitis and METS.

**Conclusion:**

Serological markers of oral pathogens were correlated with the presence and the number of METS features after multivariable adjustment. Oral bacteria acted as a mediator of the correlation between periodontitis and METS. Our study provided a biologically plausible explanation for the association between periodontitis and METS, which provides a comprehensive evaluation of periodontitis.

## Introduction

Periodontitis, a chronic inflammatory disease, affects 10–15% of the global population and results in gingival recession, alveolar bone destruction, and tooth loss (1). Dental plaque biofilms formed by periodontal microorganisms are possibly the major etiologic factors (2). Numerous periodontopathic bacteria, rather than a single periodontal pathogen, may contribute to periodontitis (2). Particular periodontal bacteria, such as *Porphyromonas gingivalis, Prevotella intermedia, Tannerella forsythia, Aggregatibacter actinomycetemcomitans*, and *Treponema denticola*, are crucial causative pathogens (3). Periodontal bacterial infection may elicit a serum immunoglobulin G (IgG) antibody response. The presence of antibodies to specific periodontal species may represent the clinical periodontal status (4).

Metabolic syndrome (METS) is an emerging public health disorder worldwide (5). Multiple factors such as sociodemographic, environmental, and genetic features influence METS (6). It is well-established that METS is associated with increased odds of cardiovascular disease, diabetes, cancer, and all-cause mortality (7). Several hypotheses, including endothelial disruption, chronic inflammation, visceral fat accumulation, and insulin resistance have been proposed as possible mechanisms (8). Endothelial dysfunction was observed in Zucker obese rats, a model of METS (9). Excessive inflammatory biomarkers were linked to the development of METS (10). Tumor necrosis factor alpha (TNF-α) may interfere with insulin signaling in human skeletal muscle (11). A case control study revealed higher interleukin-6 (IL-6) levels in subjects with METS (12). Adipokine derangement and adipocyte inflammation were implicated in the pathogenesis of METS (13). Thus, METS is considered as a chronic low-grade inflammatory status (14).

Studies reveal that periodontitis is correlated with obesity, hypertension, diabetes mellitus, and cardiovascular diseases (15–17). Several possible pathways, including endothelial dysfunction, an imbalanced immune response and oxidative stress have been presented (18, 19). Epidemiological research has examined the relationship between periodontitis and METS (20). Previous investigations indicate that periodontitis and METS are both associated with systemic inflammation, suggesting a similar pathophysiological mechanism linking these two diseases (15). However, few reports have conducted a comprehensive analysis between oral bacteria and METS. This study's objectives were to investigate the correlation between the serologic markers of 19 oral bacteria and METS and to ascertain the mediation effect of oral bacteria on the correlation between periodontitis and METS.

## Methods

### Study design and participants

Data were retrieved from the Third National Health and Nutrition Examination Survey (NHANES III) and examined for this cross-sectional study. NHANES III, an observational survey of a non-institutionalized population from the United States, was conducted by the Centers for Disease Control and Prevention and the National Center for Health Statistics between October of 1988 and October of 1994. The survey consisted of sociodemographic information, a clinical examination, medical history and laboratory data. The NHANES III survey had National Center for Health Statistics Institutional Review Board approval and details of its study protocols and consent documents were available on the NHANES website. We excluded subjects without complete information regarding the severity of periodontitis, serum IgG antibody titers against the 19 oral pathogens, the number of METS features, laboratory data, and past history. A total of 4,882 suitable subjects were initially recruited in our study.

### Definition of periodontitis

The definition and severity of periodontitis were assessed by the pocket depth and attachment loss (21). Table 1 shows the four categories of periodontitis severity.

**Table 1:** Severity of periodontitis and definition of metabolic syndrome.

**Periodontal status**	**Definition**
No	No evidence of mild, moderate, and severe periodontitis
Mild periodontitis	≥two interproximal sites with attachment loss (AL) ≥3 and <4 mm and ≥two interproximal sites with probing depth (PD) ≥4 mm not on the same tooth, or one site with PD ≥ 5 mm
Moderate periodontitis	≥two interproximal sites with AL ≥ 4 and <6 mm not on the same tooth or ≥two interproximal sites with PD ≥ 5 mm not on the same tooth
Severe periodontitis	≥two interproximal sites with AL ≥ 6 mm not on the same tooth and ≥one or more interproximal sites with PD ≥ 5 mm
**Criteria**	**Definition**
Abdominal obesity	Waist circumference ≥102 cm in men and ≥88 cm in women
Hypertriglyceridemia	≥150 mg/dL (≥1.69 mmol/L)
Reduced HDL cholesterol	<40 mg/dL (1.03 mmol/L) for men and <50mg/dL (1.29 mmol/L) for women
Elevated blood pressure	Systolic blood pressure (SBP) ≥130 mm Hg or diastolic blood pressure (DBP) ≥85 mmHg
Elevated fasting glucose	≥100 mg/dL (5.6 mmol/L)

### Serum IgG antibody measurement of oral pathogens

The level of serum IgG antibodies of 19 oral pathogens was determined using a rapid checkerboard immunoblotting technique (22). Table 2 shows the 19 oral bacterial species and strains. The bacterial IgG titers are presented as Elisa Units (EU).

**Table 2:** Nineteen periodontal bacterial species and strains.

**Periodontal bacteria species**	**Periodontal bacterial strains**
*P. gingivalis*	ATCC #33277 and #53978
*P. intermedia*	ATCC #25611
*P. nigrescens*	ATCC #33563
*T. forsythia*	ATCC #43037
*A. actinomycetemcomitans*	ATCC strains #43718, #29523, and #33384
*F. nucleatum*	ATCC #10953
*S. oralis*	ATCC #35037
*M. micros*	ATCC #33270
*C. rectus*	ATCC #33238
*E. corrodens*	ATCC #23834
*E. nodatum*	ATCC #33099
*S. intermedius*	ATCC #27335
*C. ochracea*	ATCC #33624
*V. parvula*	ATCC #10790
*A. naeslundii*	ATCC #49340
*P. melaninogenica*	ATCC #25845
*S. noxia*	ATCC #43541
*T. denticola*	OMGS #3271
*S. mutans*	ATCC #25175

### Definition of METS

The presence of METS was defined by the revised National Cholesterol Education Program's Adult Treatment Panel III (23). Table 1 lists the five components of METS. The participants were considered to have METS if they had three or more of the components.

### Covariates

Individual characteristics, including age, sex, race/ethnicity, education, smoking history, and medical records, were collected from a self-reported questionnaire. Subjects were categorized as smokers if they had smoked at least 100 cigarettes in their lifetime. We also recorded diabetes mellitus diagnosed by a doctor and measured BMI as the body weight in kilograms divided by the square of the body height in meters (kg/m^2^). Waist circumference was measured to the nearest 0.1 cm around the horizontal line at the high point of the iliac crest. We performed blood pressure measurements using a mercury sphygmomanometer. The fasting blood sugar level was quantified by a modified enzymatic reaction. The serum total cholesterol level and serum HDL cholesterol level were quantified by a Hitachi 737 analyzer (Boehringer-Mannheim Diagnostics, Indianapolis, IN). The data collection and laboratory procedures followed standardized guidelines and protocols.

### Statistical analysis

We classified the subjects based on periodontitis severity. Socio-demographic characteristics, laboratory variables, and other co-variables were compared between subjects using the one-way ANOVA and chi-square test. We examined the association between periodontitis and the number of METS features by performing multivariate regression analysis. In addition, the relationship between the IgG antibody of the oral bacteria and the number of METS features was also assessed by multivariate regression analysis. Covariates including age, sex, race/ethnicity, BMI, education, smoking, periodontitis, and diabetes mellitus, were adjusted.

We performed a mediation analysis to assess which oral bacteria mediate the relationship between periodontitis (independent variable) and METS (outcome variable) (24). Bootstrapping with 1,000 replicates was performed to calculate the medication effects without making assumptions about the normality of the sample distribution (25). The direct and indirect (mediation) effects and 95% CIs were calculated using the bias-corrected bootstrap method. If the 95% bootstrap CIs did not include zero, the indirect effect of the independent variable on the outcome variable as mediated through the intermediary was considered significant. The proportion of the indirect effect was quantified by the following formula: OR_direct effect_ (OR_indirect effect_-1)^*^100/(OR_direct effect_ × OR_indirect effect_-1) (26). All statistical analyses and data management were executed using SPSS version 18 (SPSS Inc., Chicago, IL, USA).

## Results

### Characteristics of the study population

Table 3 presents the characteristics of the participants stratified by periodontitis severity. The average age of the subjects with no, mild, moderate and severe periodontitis was 57.74 ± 13.31, 59.24 ± 13.32, 61.42 ± 13.42, and 59.99 ± 10.60 years, respectively. The SBP of the participants with no, mild, moderate and severe periodontitis was 131.15 ± 21.91, 134.43 ± 22.94, 135.87 ± 22.43, and 141.40 ± 26.20 mmHg, respectively (*p* < 0.001). The waist circumference of the participants with no, mild, moderate and severe periodontitis was 96.20 ± 13.19, 97.0 ± 12.53, 97.99 ± 13.01, and 99.48 ± 13.96 cm, respectively (*p* < 0.001).

**Table 3:** Characteristics of the study population.

**Variables**	**Periodontitis**				
	**No**	**Mild periodontitis**	**Moderate periodontitis**	**Severe periodontitis**	***p***
	**(***n*** = 3,183)**	**(***n*** = 780)**	**(***n*** = 731)**	**(***n*** = 188)**	
**Continuous variables, mean SD**					
Age (years)	57.74 (13.31)	59.24 (13.32)	61.42 (13.24)	59.99 (10.60)	<0.001
BMI (kg/m^2^)	27.81 (5.4)	27.69 (5.12)	27.73 (5.55)	28.08 (6.16)	0.820
SBP (mmHg)	131.15 (21.91)	134.43 (22.94)	135.87 (22.43)	141.40 (26.20)	<0.001
Waist circumference (cm)	96.20 (13.19)	97.0 (12.53)	97.99 (13.01)	99.48 (13.96)	<0.001
Serum total bilirubin (mg/dL)	0.58 (0.30)	0.58 (0.28)	0.58 (0.29)	0.58 (0.30)	0.991
Serum triglycerides (mg/dL)	159.79 (126.57)	159.20 (119.54)	163.03 (111.73)	175.84 (190.45)	0.36
Serum HDL cholesterol (mg/dL)	51.34 (15.58)	50 (15.38)	50.29 (17.36)	50.31 (15.57)	0.099
Serum glucose (mmol/L)	5.75 (2.23)	5.90 (2.36)	6.13 (2.65)	6.58 (3.07)	<0.001
**Periodontal pathogen antibody level (EU)**					
*P. gingivalis*	2,742 (18640.52)	4086.09 (22905.18)	7756.03 (52266.4)	10373.06 (34165.88)	<0.001
*P. intermedia*	770.36 (2698.05)	853.78 (1577.34)	960.08 (2401.22)	1125.09 (1656.58)	0.086
*P. nigrescens*	563.01 (2418.72)	498.03 (1183.87)	558.37 (1779.61)	534.7 (660.37)	0.895
*T. forsythia*	237.11 (437.26)	245.58 (339.16)	267.64 (560.01)	336.93 (553.92)	0.014
*A. actinomycetemcomitans*	3601.94 (28124.8)	3657.08 (20446.54)	4391.04 (24318.73)	3126.49 (8662.13)	0.883
*F. nucleatum*	324.06 (2600.9)	254.09 (705.86)	321.15 (973.68)	403.07 (1804.75)	0.807
*S. oralis*	180.76 (684.13)	192.26 (706.94)	181.65 (345.28)	239.70 (615.37)	0.659
*M. micros*	642.14 (3904.61)	633.41 (1901.66)	1956.02 (35928.43)	591.33 (1658.64)	0.151
*C. rectus*	258.52 (991.96)	330.69 (997.94)	401.36 (1071.95)	867.89 (2527.41)	<0.001
*E. corrodens*	394.07 (895.91)	415.05 (678.87)	452.47 (954.35)	404.20 (581.21)	0.423
*E. nodatum*	6829.22 (22596.08)	4906.48 (14203.4)	4723.98 (15782.25)	4359.09 (10881.23)	0.009
*S. intermedius*	815.49 (6728.08)	634.29 (3513.21)	844.32 (2721.27)	831.55 (2562.74)	0.87
*C. ochracea*	334.47 (1437.68)	435.71 (4603.27)	284.95 (772.49)	335.01 (729.04)	0.584
*V. parvula*	87.50 (188.38)	88.12 (144.57)	98.22 (208.56)	109.96 (189.69)	0.233
*A. naeslundii*	2220.22 (7323.02)	2104.08 (15202.25)	1707.16 (5296.69)	2133.96 (5987.80)	0.568
*P. melaninogenica*	532.61 (1759.49)	600.12 (4380.72)	493.92 (1278.36)	508.57 (714.86)	0.832
*S. noxia*	717.69 (13713.52)	300.92 (4225.41)	285.48 (3262.44)	199.82 (930.48)	0.646
*T. denticola*	390.14 (1429.60)	350.30 (792.76)	411.75 (1104.01)	592.36 (1628.43)	0.149
*S. mutans*	220.29 (904.68)	249.03 (1337.98)	210.69 (354.37)	192.50 (261.18)	0.803
**Categorical variables (%)**					
Male sex	43%	54.4%	60.5%	69.1%	<0.001
Race-ethnicity					<0.001
Non-hispanic white	55.5%	41.7%	43%	26.6%	
Education					
≥High school	63.4%	51.3%	46%	37.8%	<0.001
Smokers	13.2%	15.4%	17.5%	17%	0.011
DM	8.7%	10.4%	12.6%	20.7%	<0.001
Metabolic syndrome	44.3%	47.9%	49.7%	54.3%	0.003

*SD, standard deviation; BMI, body mass index; SBP, systolic blood pressure; HDL, high density lipoprotein; DM, diabetes mellitus; EU, Elisa units. Bold values means statistical significance*.

The serum glucose level of the participants with no, mild, moderate and severe periodontitis was 5.75 ± 2.23, 5.90 ± 2.36, 6.13 ± 2.65, and 6.58 ± 3.07 mmol/L, respectively (*p* < 0.001). The proportion of METS raised from 44.3% among subjects with no periodontitis to 47.9, 49.7, and 54.3% for participants with mild, moderate, and severe periodontitis, respectively (*p* = 0.003). The mean concentration of *P. gingivalis* IgG of the participants with no, mild, moderate, and severe periodontitis was 2,742 ± 18640.52, 4086.09 ± 22905.18, 7756.03 ± 52266.4, and 10373.06 ± 34165.88 EU, respectively (*p* < 0.001). The mean concentration of *C. recuts* IgG of the participants with no, mild, moderate and severe periodontitis was 258.52 ± 991.96, 330.69 ± 997.94, 401.36 ± 1071.95, and 867.89 ± 2527.41 EU, respectively (*p* < 0.001).

### Associations of serum IgG antibodies of oral pathogens and the number of metabolic syndrome features

Table 4 illustrates the relationship between oral pathogens and the number of metabolic syndrome features using a multivariable model. *P. nigrescens* IgG was correlated with the presence of METS (β = 192.91, *p* = 0.006). *E. corrodens* IgG was correlated with the presence of METS (β = 69.66, *p* = 0.014). *E. nodatum* IgG was correlated with the presence of METS (β = 1565.63, *p* = 0.018). *P. intermedia* IgG was correlated with the presence of METS and ≥4 features of METS (β = 277.76, *p* = 0.001; β = 633.45, *p* = 0.019, respectively). *T. forsythia* IgG was correlated with the presence of METS and ≥4 features of METS (β = 37.22, *p* = 0.011; β = 109.71, *p* = 0.025, respectively). *V. parvula* IgG was correlated with the presence of METS and ≥4 features of METS (β = 18.66, *p* = 0.002; β = 47.23, *p* = 0.02, respectively).

**Table 4.1:** Regression coefficients of the number of metabolic syndrome features for periodontal pathogens.

**Variables**		**Presence of metabolic syndrome**		**Number of metabolic syndrome features**							
				**1**		**2**		**3**		**≥4**	
		**β (95% CI)**	***p***	**β (95% CI)**	***p***	**β (95% CI)**	***p***	**β (95% CI)**	***p***	**β (95% CI)**	***p***
*P. gingivalis*	Unadjusted	2166.63 (597.53, 3735.73)	0.007	360.52 (−5370.2, 6091.25)	0.902	1741.91 (−3873.49, 7357.32)	0.543	2667.13 (−2963.48, 8297.76)	0.353	4601.16 (−1179.36, 10381.69)	0.119
	Adjusted	1434.94 (−350.71, 3220.61)	0.115	380.99 (−5347.15, 6109.14)	0.896	1309.94 (−4329.62, 6949.51)	0.649	1944.08 (−3791.56, 7679.73)	0.506	3626.46 (−2342.68, 9595.62)	0.234
*P. intermedia*	Unadjusted	257.26 (117.54, 396.97)	<0.001	166.17 (−344.15, 676.5)	0.523	190.61 (−309.44, 690.67)	0.455	369.96 (−131.45, 871.37)	0.148	545.46 (30.69, 1060.22)	0.038
	Adjusted	277.76 (118.76, 436.76)	0.001	209.64 (−300.33, 719.62)	0.42	236.38 (−265.71, 738.48)	0.356	443.58 (−67.07, 954.23)	0.089	633.45 (102.01, 1164.89)	0.019
*P. nigrescens*	Unadjusted	184.18 (63.79, 304.56)	0.003	91.08 (−348.77, 530.93)	0.685	103.37 (−327.63, 534.37)	0.638	278.83 (−153.33, 711.01)	0.206	279.89 (−163.78, 723.57)	0.216
	Adjusted	192.91 (55.65, 330.18)	0.006	115.55 (−324.87, 555.98)	0.607	124.12 (−309.49, 557.73)	0.575	307.36 (−133.63, 748.37)	0.172	319.9 (−139.05, 778.86)	0.172
*T. forsythia*	Unadjusted	51.26 (26.12, 76.41)	<0.001	50.94 (−40.9, 142.78)	0.277	66.12 (−23.86, 156.12)	0.15	100.37 (10.13, 190.61)	0.029	126.09 (33.45, 218.73)	0.008
	Adjusted	37.22 (8.58, 65.86)	0.011	54.02 (−37.85, 145.9)	0.249	61.72 (−28.72, 152.18)	0.181	88.81 (−3.17, 180.81)	0.058	109.71 (13.97, 205.45)	0.025
*A. actinomycete-mcomitans (mix)*	Unadjusted	1018.36 (−452.32, 2489.05)	0.175	1846.84 (−3525.54, 7219.22)	0.5	413.42 (−4850.84, 5677.7)	0.878	1711.63 (−3566.9, 6990.17)	0.525	2322.43 (−3096.63, 7741.51)	0.401
	Adjusted	1484.24 (−192.51, 3161.01)	0.083	1948.71 (−3430.38, 7327.81)	0.478	673.04 (−4622.87, 5968.95)	0.803	2286.83 (−3099.3, 7672.96)	0.405	3015.49 (−2589.92, 8620.91)	0.292
*F. nucleatum*	Unadjusted	98.17 (−25.4, 221.74)	0.119	126.07 (−325.21, 577.35)	0.584	64.02 (−378.18, 506.22)	0.777	109.93 (−333.47, 553.33)	0.627	313.87 (−141.33, 769.08)	0.177
	Adjusted	125.94 (−14.99, 266.87)	0.08	150.58 (−301.37, 602.55)	0.514	109.41 (−335.55, 554.38)	0.63	179.24 (−273.3, 631.79)	0.437	397.93 (−73.04, 868.9)	0.098

*Bold values means statistical significance*.

**Table 4.2:** Regression coefficients of the number of metabolic syndrome features for periodontal pathogens.

**Variables**		**Presence of metabolic syndrome**		**Number of metabolic syndrome features**							
				**1**		**2**		**3**		**≥4**	
		**β (95% CI)**	***p***	**β (95% CI)**	***p***	**β (95% CI)**	***p***	**β (95% CI)**	***p***	**β (95% CI)**	***p***
*S. oralis*	Unadjusted	31.69 (−4.89, 68.28)	0.09	52.05 (−81.61, 185.73)	0.445	30.48 (−100.49, 161.47)	0.648	62.27 (−69.06, 193.61)	0.353	80.5 (−54.33, 215.33)	0.242
	Adjusted	19.27 (−22.42, 60.97)	0.365	54.34 (−79.43, 188.12)	0.426	28.67 (−103.03, 160.38)	0.67	51.6 (−82.34, 185.56)	0.45	61.28 (−78.11, 200.69)	0.389
*M. micros*	Unadjusted	−477.13 (−1286.42, 332.15)	0.248	288.83 (−2667.91, 3245.58)	0.848	743.76 (−2153.49, 3641.01)	0.615	70.09 (−2835,01, 2975.19)	0.962	86.64 (−2895.8, 3069.09)	0.955
	Adjusted	−297.97 (−1220.1, 624.15)	0.526	444.02 (−2514.43, 3402.47)	0.769	1021.56 (−1891.14, 3934.26)	0.492	490.11 (−2472.21, 3452.44)	0.746	623.75 (−2459.17, 3706.68)	0.692
*C. rectus*	Unadjusted	72.98 (10.45, 135.52)	0.022	49.29 (−179.1, 277.69)	0.672	100.68 (−123.12, 324.48)	0.378	127.09 (−97.31, 351.5)	0.267	196.25 (−34.13, 426.63)	0.095
	Adjusted	24.93 (−46.05, 95.92)	0.491	64.12 (−163.62, 291.86)	0.581	85.57 (−138.64, 309.79)	0.454	85.74 (−142.29, 313.78)	0.461	140.46 (−96.86, 377.78)	0.246
*E. corrodens*	Unadjusted	62.15 (13.48, 110.82)	0.012	2.46 (−175.33, 180.25)	0.978	20 (−154.21, 194.21)	0.822	55.26 (−119.42, 229.95)	0.535	111.6 (−67.73, 290.94)	0.223
	Adjusted	69.66 (14.34, 124.98)	0.014	9.22 (−168.21, 186.66)	0.919	20.93 (−153.76, 195.62)	0.814	66.12 (−111.54, 243.79)	0.466	135.44 (−49.45, 320.35)	0.151
*E. nodatum*	Unadjusted	563.52 (−578.26, 1705.31)	0.333	768.75 (−3402.97, 4940.47)	0.718	829.62 (−3258.14, 4917.4)	0.691	1467.42 (−2631.42, 5566.28)	0.483	1105.22 (−3102.75, 5313.19)	0.607
	Adjusted	1565.63 (271.59, 2859.67)	0.018	1207.84 (−2943.68, 5359.37)	0.568	1958.61 (−2128.71, 6045.93)	0.348	3282.65 (−874.3, 7439.61)	0.122	3187.19 (−1139, 7513.39)	0.149
*S. intermedius*	Unadjusted	283.35 (−41.27, 607.99)	0.087	−126.23 (−1311.53, 1050.07)	0.835	10.5 (−1150.94, 1171.95)	0.986	13.67 (−1150.92, 1178.26)	0.982	668.71 (−526.89, 1864.31)	0.273
	Adjusted	302.26 (−67.88, 672.41)	0.109	−51.63 (−1238.43, 1135.16)	0.932	119.64 (−1048.8, 1288.08)	0.841	169.23 (−1019.11, 1357.58)	0.78	845.57 (−391.15, 2082.29)	0.18

*Bold values means statistical significance*.

**Table 4.3:** Regression coefficients of the number of metabolic syndrome features for periodontal pathogens.

**Variables**		**Presence of metabolic syndrome**		**Number of metabolic syndrome features**							
				**1**		**2**		**3**		**≥4**	
		**β (95% CI)**	***p***	**β (95% CI)**	***p***	**β (95% CI)**	***p***	**β (95% CI)**	***p***	**β (95% CI)**	***p***
*C. ochracea*	Unadjusted	18.67 (−106.02, 143.37)	0.769	99.88 (−355.57, 555.33)	0.667	139.13 (−307.15, 585.42)	0.541	77.14 (−370.34, 524.64)	0.735	251.04 (−208.36, 710.45)	0.284
	Adjusted	3.87 (−138.39, 146.13)	0.957	90.42 (−365.9, 546.75)	0.698	128.43 (−320.84, 577.7)	0.575	67.4 (−389.52, 524.33)	0.772	237.56 (−237.96, 713.09)	0.327
*V. parvula*	Unadjusted	18.04 (7.62, 28.46)	0.001	11.15 (−26.9, 49.21)	0.566	21.33 (−15.95, 58.62)	0.262	31.52 (−5.87, 68.91)	0.099	41.19 (2.8, 79.58)	0.035
	Adjusted	18.66 (6.79, 30.53)	0.002	13.16 (−24.91, 51.23)	0.498	24.28 (−13.19, 61.77)	0.204	35.66 (−2.46, 73.78)	0.067	47.23 (7.55, 86.9)	0.02
*A. naeslundii*	Unadjusted	−237.21 (−735.02, 260.59)	0.35	−577.16 (−2395.52,1241.19)	0.534	−958.5 (−2740.27, 823.26)	0.292	−893.83 (−2680.42, 892.76)	0.327	−1260.15 (−3094.31, 574)	0.178
	Adjusted	90.93 (−475.71, 657.57)	0.753	−484.32 (−2302.29, 1333.64)	0.601	−668.57 (−2458.42, 1121.28)	0.464	−424.97 (−2245.31, 1395.37)	0.647	−698.82 (−2593.28, 1195.63)	0.47
*P. melaninogenica*	Unadjusted	104.37 (−26.6 235.34)	0.118	111.86 (−366.52, 590.25)	0.647	127.16 (−341.6, 595.92)	0.595	157.79 (−312.24, 627.82)	0.51	337.6 (−144.94, 820.14)	0.17
	Adjusted	113.64 (−35.78, 263.06)	0.136	126.15 (−353.09, 605.4)	0.606	149.19 (−322.65, 621.03)	0.535	194.96 (−284.92, 674.84)	0.426	387.61 (−111.8, 887.02)	0.128
*S. noxia*	Unadjusted	−453.49 (−1092.44, 185.46)	0.164	1173.24 (−1160.71, 3507.2)	0.324	489.57 (−1797.41, 2776.57)	0.675	192.92 (−2100.27, 2486.11)	0.869	386.24 (−1967.99, 2740.49)	0.748
	Adjusted	−332.15 (−1051.02, 406.72)	0.386	1213.04 (−1125.13, 3551.22)	0.309	618.14 (−1683.87, 2920.17)	0.599	384.45 (−1956.78, 2725.69)	0.748	600.53 (−1836.02, 3037.08)	0.629
*T. denticola*	Unadjusted	3.93 (−70.3, 78.18)	0.917	241.06 (−30.02, 512.15)	0.081	120.87 (−144.75, 386.51)	0.372	165.99 (−100.35, 432.34)	0.222	157.62 (−115.82, 431.06)	0.259
	Adjusted	12.55 (−71.85, 96.96)	0.771	267.17 (−3.47, 537.83)	0.053	147.42 (−119.04, 413.88)	0.278	194.71 (−76.28, 465.72)	0.159	178.85 (−103.18, 460.89)	0.214
*S. mutans*	Unadjusted	45.99 (−5.94, 97.92)	0.083	29.84 (−159.87, 219.55)	0.758	79.56 (−106.33, 265.46)	0.401	98.51 (−87.88, 284.91)	0.3	116.27 (−75.08, 307.63)	0.234
	Adjusted	26.36 (−32.86, 85.58)	0.383	33.79 (−156.19, 223.78)	0.727	78.8 (−108.24, 265.86)	0.409	87.57 (−102.66, 277.81)	0.367	96.03 (−101.95, 294.01)	0.342

*Adjusted variables = age, gender, race-ethnicity, education, body mass index, smoking, diabetes mellitus and periodontitis. β, coefficient; CI, confidence interval; p, p-value. Bold values means statistical significance*.

### Mediation of serum IgG antibodies of oral pathogens for the association between periodontitis and METS

Table 5 presents the significant indirect effect of periodontitis on METS as only mediated through *P. intermedia* IgG (β = 0.003; 95% CI: 0.0007, 0.01). The proportion mediated through *P. intermedia* IgG was 11.2%. The indirect effects mediated through the other five oral bacterial antibodies were not significant, as their 95% CIs included zero.

**Table 5:** Mediation of serum IgG antibodies of oral bacteria for the association between periodontitis and metabolic syndrome.

**Pathway**		***P. intermedia***					***P. nigrescens***				
		**β**	**95% CI**	**OR**	**95% CI**	***p***	**β**	**95% CI**	**OR**	**95% CI**	***p***
X → M		0.059	0.033, 0.086			<0.001	0.005	−0.021, 0.031			0.719
M|X → Y		0.05	0.021, 0.007			0.001	0.065	0.036, 0.093			<0.001
X|M → Y	(direct effect)	0.032	0.003, 0.06	1.032	0.96, 1.09	0.02	0.034	0.006, 0.06	–	–	0.016
X → M → Z	(indirect effect)	0.003	0.0007, 0.01	1.003	0.99, 1.008	0.004	0.0006	−0.002, 0.0001	–	–	0.92
Percent of mediation effect		11.2%					No mediation				
**Pathway**		***T. forsythia***					***E. corrodens***				
		**β**	**95% CI**	**OR**	**95% CI**	***p***	**β**	**95% CI**	**OR**	**95% CI**	***p***
X → M		0.036	0.01, 0.065			0.011	0.022	−0.002, 0.048			0.086
M|X → Y		0.038	0.009, 0.064			0.006	0.041	0.014, 0.068			0.004
X|M → Y	(direct effect)	0.033	0.004, 0.06	–	–	0.016	0.033	0.006, 0.06	–	–	0.01
X → M → Z	(indirect effect)	0.001	−0.00003, 0.0001	–	–	0.06	0.0009	−0.0006, 0.0001	–	–	0.254
Percent of mediation effect		No mediation					No mediation				
**Pathway**		***E. nodatum***					***V. parvula***				
		**β**	**95% CI**	**OR**	**95% CI**	***p***	**β**	**95% CI**	**OR**	**95% CI**	***p***
X → M		−0.038	−0.062, −0.015			0.002	0.014	−0.012, 0.04			0.271
M|X → Y		0.021	−0.006, 0.047			0.13	0.026	−0.001, 0.055			0.062
X|M → Y	(direct effect)	0.035	0.009, 0.06	–	–	0.01	0.034	0.007, 0.06	–	–	0.01
X → M → Z	(indirect effect)	−0.001	−0.002, 0.0001	–	–	0.2	0.0005	−0.0005, 0.0001	–	–	0.374
Percent of mediation effect		No mediation					No mediation				

*b, coefficient; CI, confidence interval; p, p-value; OR, Odds ratio; X, independent variable (Periodontitis); M, mediator (Periodontal bacteria); Y, outcome variable (metabolic syndrome). **X** **→ M**, effect of X on M; **M| X** **→ Y**, effect of M on Y controlling for X; **X| M** **→** **Y**, effect of X on Y controlling for M (direct effect); **X → M → Y**, effect of X on Y mediated by M (indirect effect)*.

## Discussion

This study draws attention to the association between serum IgG antibodies of oral pathogens and the number of METS features. We observed that the antibodies of two periodontal bacterial (*P. gingivalis* and *C. recuts*) increased with the severity of periodontitis in a univariate analysis (Table 3). Participants with more severe periodontitis had a higher proportion of METS compared to those without periodontitis. We demonstrated a positive relationship between the antibodies of *P. nigrescens, E. corrodens, E. nodatum*, and the presence of METS. The antibodies of *P. intermedia, T. forsythia*, and *V. parvula* were positively correlated with the presence of METS and ≥4 features of METS. We observed an indirect effect between periodontitis and METS through *P. intermedia* IgG in the mediation analysis.

Previous literature revealed that periodontal bacteria was associated with the component of METS. A Japanese cross-sectional study reported that an elevated level of antibodies against *P. gingivalis* was observed in METS (27). Periodontal bacterial antibody (*P. gingivalis* and *P. intermedia*) titers were correlated with elevated blood glucose levels (28). The Oral Infections and Vascular Disease Epidemiology Study noted that compared with the lowest subgingival periodontal bacterial burden, subjects with the highest subgingival periodontal bacterial burden had a three times greater chance of having hypertension (29). An observational study from Columbia demonstrated antibodies of periodontopathic bacteria were associated with a reduced level of HDL cholesterol (30). In addition, serologic markers of periodontal pathogens are correlated with an elevated odd of stroke (31), type 2 diabetes mellitus (32), and coronary heart disease (33).

Studies have suggested that periodontal bacteria may induce an immune response and inflammatory processes (34). The serum IgG antibodies against *P. gingivalis, P. intermedia*, and *E. corrodens* are linked to oxidative stress (35). The local infections caused by periodontal bacteria were associated with systemic inflammatory markers including C-reactive protein (CRP), IL-6, and TNF-α (36, 37). Several plausible mechanisms have been proposed. Lipopolysaccharide, a constituent of the outer membrane of periodontopathogenic bacteria such as *P. intermedia, T. forsythia*, and *V. parvula* has been indicated to stimulate the production of various proinflammatory mediators (38–40). In an experimental study by Sun et al. (41), lipopolysaccharides elicited the release of inflammatory cytokines through toll-like receptors 2 and 4. Furthermore, complement system activation and neuropeptide modulation are linked to inflammation (42). Complement receptor 3 (CR3) activation by periodontal bacterial fimbriae and C5a accumulation may result in increased inflammation (43). Substance P, a neuropeptide, is involved in periodontal inflammation process (44). Collectively, these results suggest that periodontal bacterial infection could cause immune dysregulation (45) and result in systemic inflammation (1). Systemic inflammation plays an essential role in the pathogenesis of METS (46). Oxidative stress was also recognized as a potential pathophysiological link to the association between METS and periodontitis (47). Although the mechanism underlying the relationship between periodontal bacteria and METS remains unclear, the abovementioned investigations indicate that periodontal infection and subsequent inflammation might increase the risk of METS.

In this study, we discovered a mediation effect of *P. intermedia* on the association between periodontitis and METS. Our analyses imply that periodontal bacteria antibodies play a modest role in the mediation analysis. Prior reports have indicated the mediation effect of systemic inflammation regarding periodontitis. Demmer et al. (48) reported that inflammatory markers act as a mediator in the association between periodontal infection and insulin resistance. A cross-sectional study from Thailand revealed CRP (5.2%) and white blood count (19.1%) are mediators of the relationship between periodontitis and impaired fasting glucose (49). The significant indirect effect of periodontal infection on hypertension mediated through CRP was determined *via* two national databases (50).

There are several limitations in the present investigation. The cross-sectional analysis restricts the causal relationship between oral bacteria and METS. Our data were retrieved from a single database, and the study population was mainly Caucasian. Thus, the generalization of our results is limited. Lastly, because of the nature of observational studies, our results will inevitably be influenced by residual confounding factors due to unmeasured covariates.

## Conclusion

Our study extended the examinations of the commonly reported relationship between *P. gingivalis* and *P. intermedia* to 19 oral bacterial antibodies. The serum IgG antibodies of oral pathogens were correlated with the presence and the number of METS features. We also conducted a mediation analysis, which revealed that *P. intermedia* acts as a mediator in the correlation between periodontitis and METS. An experimental model and prospective study are warranted to investigate the mechanisms underlying the observed correlations and to explore the potential median effect of oral bacteria.

## Data availability statement

The original contributions presented in the study are included in the article/supplementary material, further inquiries can be directed to the corresponding author/s.

## Ethics statement

The studies involving human participants were reviewed and approved by the National Center for Health Statistics Research Ethics Review Board. The patients/participants provided their written informed consent to participate in this study. Written informed consent was obtained from the individual(s) for the publication of any potentially identifiable images or data included in this article.

## Author contributions

Z-YY: conceptualization, investigation, visualization, and writing—original draft preparation. W-HF: methodology, formal analysis, and supervision. C-CK: investigation and writing—original draft preparation. W-LC: conceptualization, data curation, methodology, formal analysis, supervision, and writing—review and editing. All authors have read and agreed to the published version of the manuscript.

## Conflict of interest

The authors declare that the research was conducted in the absence of any commercial or financial relationships that could be construed as a potential conflict of interest.

## Publisher's note

All claims expressed in this article are solely those of the authors and do not necessarily represent those of their affiliated organizations, or those of the publisher, the editors and the reviewers. Any product that may be evaluated in this article, or claim that may be made by its manufacturer, is not guaranteed or endorsed by the publisher.

## Abbreviations

METS: metabolic syndrome
BMI: body mass index
CRP: c-reactive protein
IL-6: interleukin-6
TNF-α: tumor necrosis factor-α
IgG: immunoglobulin G
CR3: complement receptor 3
EU: elisa units
NCHS: National Center for Health Statistics
NHANES III: the third National Health and Nutrition Examination Survey
HDL: high density lipoprotein
SBP: systolic blood pressure.
